# Non-invasive derivation of instantaneous free-wave ratio from invasive coronary angiography using a new deep learning artificial intelligence model and comparison with human operators’ performance

**DOI:** 10.1007/s10554-025-03369-y

**Published:** 2025-03-10

**Authors:** Catarina Oliveira, Marta Vilela, João Silva Marques, Cláudia Jorge, Tiago Rodrigues, Ana Rita Francisco, Rita Marante de Oliveira, Beatriz Silva, João Lourenço Silva, Arlindo L. Oliveira, Fausto J. Pinto, Miguel Nobre Menezes

**Affiliations:** 1 Structural and Coronary Heart Disease Unit, Cardiovascular Center of the University of Lisbon (CCUL@RISE), Faculdade de Medicina, Universidade de Lisboa, Serviço de Cardiologia, Avenida Professor Egas Moniz, Lisboa, 1649-028 Portugal; 2 Serviço de Cardiologia, Departamento de Coração e Vasos, CHULN Hospital de Santa Maria, Av Prof. Egas Moniz, Lisboa, 1649-028 Portugal; 3 Faculdade de Medicina, Universidade de Lisboa, Av Prof. Egas Moniz, Lisboa, 1649-028 Portugal; 4 INESC-ID, Instituto Superior Técnico, Universidade de Lisboa, Av. Rovisco Pais, Lisboa, 1000-049 Portugal; 5 Neuralshift, Inc. Av. Duque d’Ávila 23, Lisboa, 1000 − 138 Portugal

**Keywords:** Artificial intelligence, Coronary physiology, Instantaneous free-wave ratio, Coronary artery disease

## Abstract

Invasive coronary physiology is underused and carries risks/costs. Artificial Intelligence (AI) might enable non-invasive physiology from invasive coronary angiography (CAG), possibly outperforming humans, but has seldom been explored, especially for instantaneous wave-free Ratio (iFR). We aimed to develop binary iFR lesion classification AI models and compare them with human performance. single-center retrospective study of patients undergoing CAG and iFR. A validated encoder-decoder convolutional neural network (CNN) performed segmentation. Manual annotation of target vessel and pressure sensor location on a segmented telediastolic frame followed. Three AI models classified lesions as positive (≤ 0.89) or negative (> 0.89). Model 1 uses preprocessed vessel diameters with a transformer. Models 2/3 are EfficientNet-B5 CNNs using concatenated angiography and segmentation - Model 3 employs class-frequency-weighted Cross-Entropy Loss. Previous findings demonstrated Model 3’s superiority for left anterior descending (LAD) and Model 1’s for circumflex (Cx)/right coronary artery (RCA) - they were therefore unified into a vessel-based model. Ten-fold patient-level cross-validation enabled full sample training/testing. Three experienced operators performed binary iFR classification using single frames of raw/segmented images. Comparison metrics were accuracy, sensitivity, specificity, positive predictive value (PPV) and negative predictive value (NPV). Across 250 measurements, AI accuracy was 72%, PPV 48%, NPV 90%, sensitivity 77%, and specificity 71%. Human accuracy ranged from 54 to 74%. NPV was high for the Cx/RCA (AI: 96/98%; operators: 94/97%), but AI significantly outperformed humans in the LAD (78% vs. 60–64%). An AI model capable of binary iFR lesions classification mildly outperformed interventional cardiologists, supporting further validation studies.

**Supplementary Information:**

The online version contains supplementary material available at 10.1007/s10554-025-03369-y.

## Introduction

Coronary angiography remains the cornerstone for the evaluation and treatment of coronary artery disease (CAD) [1]. However, it presents major limitations as a method to ascertain the functional relevance of coronary stenoses, especially in lesions of intermediate angiographic severity [1]. Invasive coronary physiology has been extensively studied to partly overcome these limitations and is recommended in clinical guidelines [2, 3]. However, despite initial positive landmark clinical trials [4–7]_,_ the impact of physiology in more complex clinical scenarios (such as acute coronary syndrome and three vessel disease [8–9] has not been unequivocally demonstrated, likely due to the limitations of physiology imposed by the acute setting and/or the risk of complications.

Fractional flow reserve (FFR) is the most widely studied pressure-derived index^[1^. Over the last few years, another index gained importance– instantaneous free-wave ratio (iFR) - and its equivalent diagnostic accuracy comparing to FFR has been demonstrated [10–16]. Furthermore, iFR is simpler to use (given it does nor require adenosine) and may be better suited for tandem lesions as well as post-PCI physiology prediction [10–16].

Despite the established evidence, coronary physiology is largely underused − 3 to 30% in PCI procedures [17, 18]. The aforementioned risk of complications and challenges associated with technical aspects of the procedure are likely reasons for this [17–18].

Non-invasive derivation of coronary physiology from Coronary angiography (CAG) may partly overcome these limitations, by removing the invasive component of measurents (and its associated risks) while simultaneously potentially increase the adoption of physiology [19–21].While such digitally-derived indexes have recently been developed, a number of issues remain. First, they are semi-automatic (i.e. require manual input) and are therefore prone to operator variability, especially outside a core lab [20–21]. Second, they have focused solely on FFR– given that no single index is desirable, exploring the deriation of iFR instead is scientifically and clinically desirable [20–21]. Third, they have resorted primarily to traditional computer methods, namely computational fluid dynamics [20–21] - more recent Artificial intelligence (AI) methods have seldom been explored [22]. Fourth, while some software has reached a significant level of maturity, namely QFR, the recent negative results of the FAVOR-3 Europe trial [23] - where QFR was inferior to invasive FFR - clearly highlight that further research is necessary.

Artificial intelligence (AI) has already shown great potential in Cardiovascular Imaging and is currently boosting the development of interventional solutions that may pave the way to overcome some of the above mentioned limitations [22, 24–30]. Transthoracic echocardiography (TTE) is the most widely used imaging modality in cardiology for evaluating cardiac structure and function [29]. Nevertheless, operator-dependent discrepancies can impact the precision of TTE assessments [29]. AI has emerged as a transformative tool in this domain, enabling automated segmentation of cardiac chambers (and consequently allowing for volumes and mass measurements) as well as longitudinal strains determination. In addition to automated analysis, AI has shown promising results for the classification or diagnosis of several cardiac pathologies [25, 26, 29] and the uniformisation of procedures markedly reduces this interoperator (and intraoperator) variability [29]. AI is also revolutionizing the diagnostic and prognostic capabilities in the field of cardiac computed tomography (CT), improving imaging quality and post-processing, with significant impact in coronary artery disease accurate detection [29, 31]. Deep learning algorithms have also been developed to detect high-risk lesions, enabling evaluation of atherosclerotic burden, calcification, plaque thickness and severity of stenosis [32]. AI technologies are also revolutionizing cardiovascular magnetic resonance (CMR) and has mainly been use for segmentation of cardiac structures and infarct size [27–29].

Regarding interventional cardiology, novel reconstruction procedures have been applied to intravascular imaging and to dynamic coronary roadmap [22]. Nonetheless, AI use regarding coronary physiology is still rudimentary [33], and its implementation in clinical practice could overcome some limitations, as the need to use invasive maneuvers to access coronary physiology, while also increasing physiology adoption.

We have recently developed fully automated AI models capable of automatic CAG segmentation and binary iFR lesion classification [33, 35]. In this study, we aimed to compare their performance with the binarily iFR classification of three experienced interventional cardiologists, regarding accuracy, sensitivity, specificity and positive and negative predictive value.

## Methods

### Patient population, inclusion and exclusion criteria

This was a single center retrospective selection of consecutive patients in a three-year period (2017 to 2019), who had undergone both coronary angiography (CAG) and invasive physiology assessment with iFR (Philips Volcano System), regardless of clinical context (i.e. both acute and chronic coronary syndrome).

We excluded cases where any of the following was present:

1. Imaging criteria:

- Patients with cardiac devices or other sources of potential imaging artifacts overlapping with the coronary tree image.
- Poor image quality.
- Unsuccessful segmentation with AI models.
- Unclear individualization of lesion outline with overlapping vessels.

1. Clinical criteria.
  - History of coronary artery bypass surgery (CABG) or valvular intervention (surgical or percutaneous).
  - Significant left heart valvular disease (severe aortic stenosis or regurgitation, severe mitral regurgitation, moderate or severe mitral stenosis, moderate valvular disfunction in both aortic and mitral valves).
  - Target culprit vessel culprit of acute coronary syndrome (ACS).
  - Target vessel non-culprit of ACS in patient with ST segment elevation myocardial infarction (STEMI) within 48 h of presentation.
  - Previous transmural myocardial infarction in target-vessel.
  - Chronic total occlusion not previous treated with PCI in any vessel.
  - Left ventricular systolic disfunction, defined as an ejection fraction < 50%.
  - Cardiogenic shock.
  - Haemodynamic instability.
  - Left main lesions.

b. These criteria were selected because of their potential impact on iFR measurements, thereby potentially confounding both AI models and/or operators.

### AI models development and lesion assessment

We first created AI models capable of fully automated segmentation of CAG images and published the associated data [35, 36]. Subsequently, we created three AI models capable of binary iFR classification [36]. Here, we briefly summarize the methods and architecture of the models.

For segmentation, we utilized an encoder-decoder fully convolutional neural network based on the U-Net, a common choice for medical image segmentation. To determine the most effective approach, we conducted a comparative analysis of encoder and decoder architectures. This led to the proposal of EfficientUNet++, a computationally efficient and high-performing decoder architecture, which we paired with an EfficientNet-B5 encoder in this work. We trained our segmentation model using patients who underwent CAG and invasive physiology assessments with at least intermediate lesions in one or more vessels were retrospectively and randomly selected, with a total of 416 images. We then validated our segmentation models in multicentric fashion [35, 36].

**Fig. 1:**
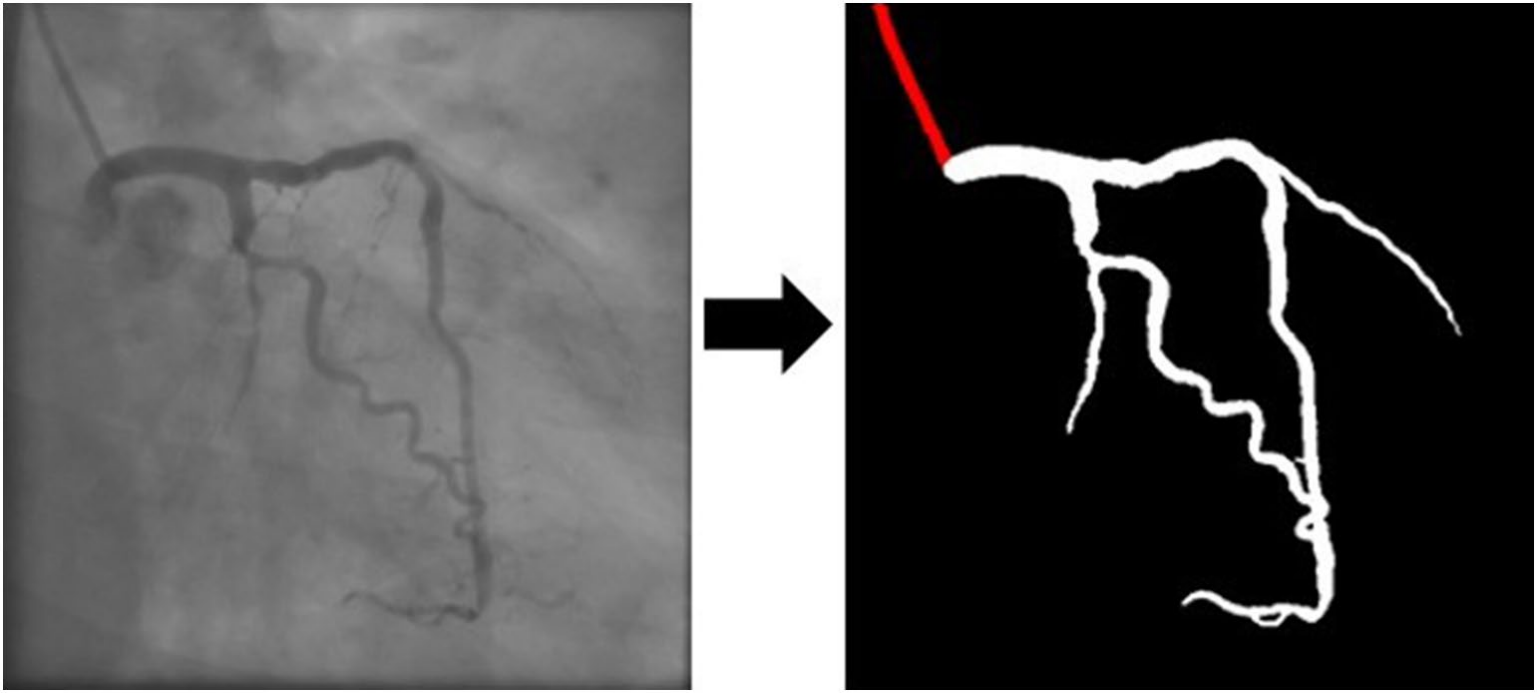
Artificial intelligence (AI) automatic segmentation from coronary angiography images

For the iFR classification models, we began by automatically segmenting the angiograms using our previously developed model (Fig. 1). We then proceeded to manually annotate the target vessel and the location of the pressure sensor wires used for iFR measurements, focusing on a single telediastolic frame where the target vessel was outlined. Initial tests of the AI models revealed improved performance when a single image, clearly outlining the target vessel, was used, rather than multiple projections. Consequently, our training approach focused on this single-frame selection. Three models were trained, utilizing the CAG images in both their original greyscale form and manually annotated versions produced after automatic segmentation. The models were trained to classify targets binarily based on iFR values: ≤ 0.89 (positive) or > 0.89 (negative). To ensure robust evaluation, the models were tested on data not used during training. A cross-validation approach was applied at patient level while maintaining the distribution of target vessels and iFR classifications across splits. Each subset’s iFR classification was conducted using neural networks trained exclusively on the remaining data, allowing for comprehensive performance evaluation across the entire cohort. This avoided the limitations of a fixed train/test split, which would have reduced the size of the testing group and limited performance assessment.

A total of 3 models capable of binary iFR classification were developed, with varying degrees of performance according to target vessel. Model 1 used as input the sequence of diameters of the target vessel, automatically preprocessed by a custom script. This model then processes the data using a transformer encoder. Models 2 and 3 are Convolutional Neural Networks (CNNs), based on EfficientNet-B5, which took as input the concatenation of the single-channel angiography image and its segmentation. They both used a simple Cross-Entropy Loss, but model 3 is weighted by the inverse of each iFR class’s frequency, in order to mitigate the negative effects of class imbalance. In our previous work, we have found model 3 to deal best with the left anterior descending artery and model 1 to deal best with circumflex and right coronary artery lesions [36]. As a result, we have unified them into a single best-performing model, where the model is selected according to the target vessel.

### Lesion assessment by interventional cardiologists

Three interventional cardiologists (experience ranging from 7 to 12 years) were asked to classify the target lesions binarily based on two images: a single “raw” fluoroscopic frame and a single frame entirely segmented by the AI models, thereby mimicking what the models actually “see”.

We have previously shown the segmentation produced by our models to be highly accurate when compared to the fluoroscopy images [34–36]. Therefore, disagreements between raw and segmented images were assumed to be due to the perception effect on the operators visual estimation of stenosis [36], rather than true discrepancies between vessel dimensions in the raw vs. segmented images.

### Performance assessment and statistical analysis

As previously described in detail [36] we used a cross-validation split method for training and testing our models, in order to ensure our testing sample was large enough for reaching a meaningful assessment. By splitting the data at a patient level in 10 subsets, while retaining the relative distribution of target vessel and iFR classification, we were able use the full sample for testing. We had already used this approach in the past for developing our segmentation models [34–36].

Descriptive variables are shown in absolute and relative (percentage) numbers. Quantitative variables are shown in average ± standard deviation (if normally distributed) or median (interquartile range) if non-normally distributed. The chi-square test was used for statistically comparing the binary classification of measured iFR vs. that of the models vs. that classified by the operators. A p-value < 0.05 was used for statistical significance.

The results of the models’ classification of target lesions as either iFR positive or negative were compared to those of the true (i.e. real) invasive iFR measurements, as follows:

- True positive (TP): both estimated and real iFR were positive.
- False positive (FP): positive estimated iFR and negative real iFR.
- True negative (TN): both estimated and real iFR were negative.
- False negative (FN): negative estimated iFR and positive real iFR.

Using this classification, the following parameters were calculated:

- Accuracy: ([TP + TN]/[TP + TN + FP + FN])
- Sensitivity: TP / (TP + FN).
- Specificity: TN/(TN + FP),
- Positive Predictive Value (PPV): TP / (TP + FP).
- Negative predictive value (NPV): TN/(TN + FN).

In addition to these metrics, we also performed a modified ROC curve analysis, for two reasons. First, physiology datasets are often significantly imbalanced towards negative (iFR > 0.89) results and therefore accuracy as calculated by the above-mentioned metrics may not reflect the discriminatory ability to classify lesions. Second, identifying and correctly classifying negative lesions in this clinical context may arguably be of greater importance, as it would enable procedure termination without engaging in additional testing, which includes engaging in further maneuvers (guide catheters, wiring the vessel, additional heparin, adenosine administration). By contrast, a positive result is likely to require either additional invasive testing and/or revascularization and would therefore require at least a part of said additional steps. Furthermore, correctly identifying a negative result may also limit inappropriate revascularization. Thus, we plotted the true iFR > 89 rate (i.e. true negative rate) vs. the false iFR > 0.89 rate (i.e. false negative rate) and calculated the AUC, instead of the typical true positive vs. false positive rate. By doing so we prioritized the discriminatory ability for detecting iFR lesions > 0.89 rather than ≤ 0.89.

SPSS 27 and dedicated python scripts were used for analysis.

### Ethical issues

This study complies with the Declaration of Helsinki and was approved by the local Ethics’ Institutional Review Board.

## Results

### Baseline characteristics

A total of 334 patients were screened. Considering the exclusion criteria, 250 measurements, from 223 patients (age 68 ± 11 years old, 66.37% male), were included - Table 1. iFR was performed predominantly in a chronic setting (66.37%). Left descending coronary artery (LAD) was the most evaluated vessel (51.6%), followed by right coronary artery (RCA) and circumflex (Cx)– Table 2. Most lesions had an iFR > 0.89, with the majority of right and circumflex coronary arteries lesions with a negative iFR. The difference was much less pronounced in the left anterior descending artery measurements, with 42.5% of positive iFR values.

**Table 1:** Clinical characteristics of included patients

Parameter	*N* +/- SD or *N*(%)
Age	68 ± 11
Sex (male)	148 (66.37%)
Hypertension	180 (80.72%)
Diabetes mellitus	96 (43.05%)
Dyslipidemia	132 (59.19%)
Smoker (past or present)	89 (39.91%)
Chronic coronary syndrome	148 (66.37%)
Acute coronary syndrome	74 (33.18%)

**Table 2:** iFR results overall and stratified per artery. LAD– Left anterior descending artery. RCA– Right coronary artery; CX– Circumflex artery. SD– Standard deviation

	*N* (%)	iFR (mean ± SD)	iFR ≤ 0.89 (*N* / %)	iFR > 0.89 (*N* / %)
Total	250 (100%)	0,91 ± 0,006	65 (26.0%)	185 (74.0%)
LAD	129.0 (51.6%)	0,88 ± 0,009	55 ( 42,5%)	74 (57,4%)
RCA	76.0 (30.4%)	0,95 ± 0,009	5 (6,6%)	71 (93,4%)
CX	45.0 (18.0%)	0,96 ± 0,009	5 (33.33%)	40 (88,9%)

### Comparison of AI and interventional cardiologists performance

### All arteries

**Fig. 2:**
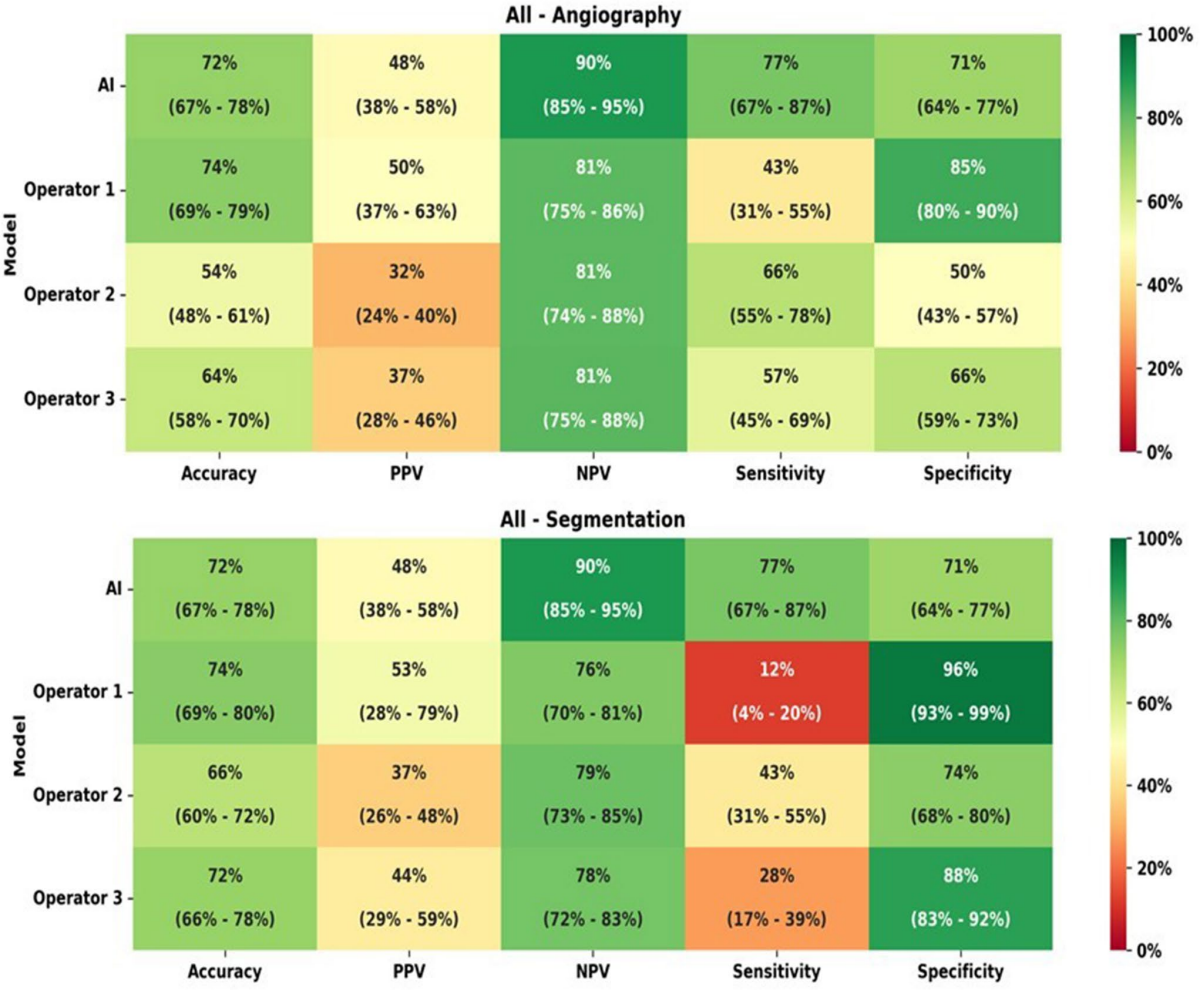
Heatmap analyzes for all arteries, regarding both angiography images and segmentation

**Fig. 3:**
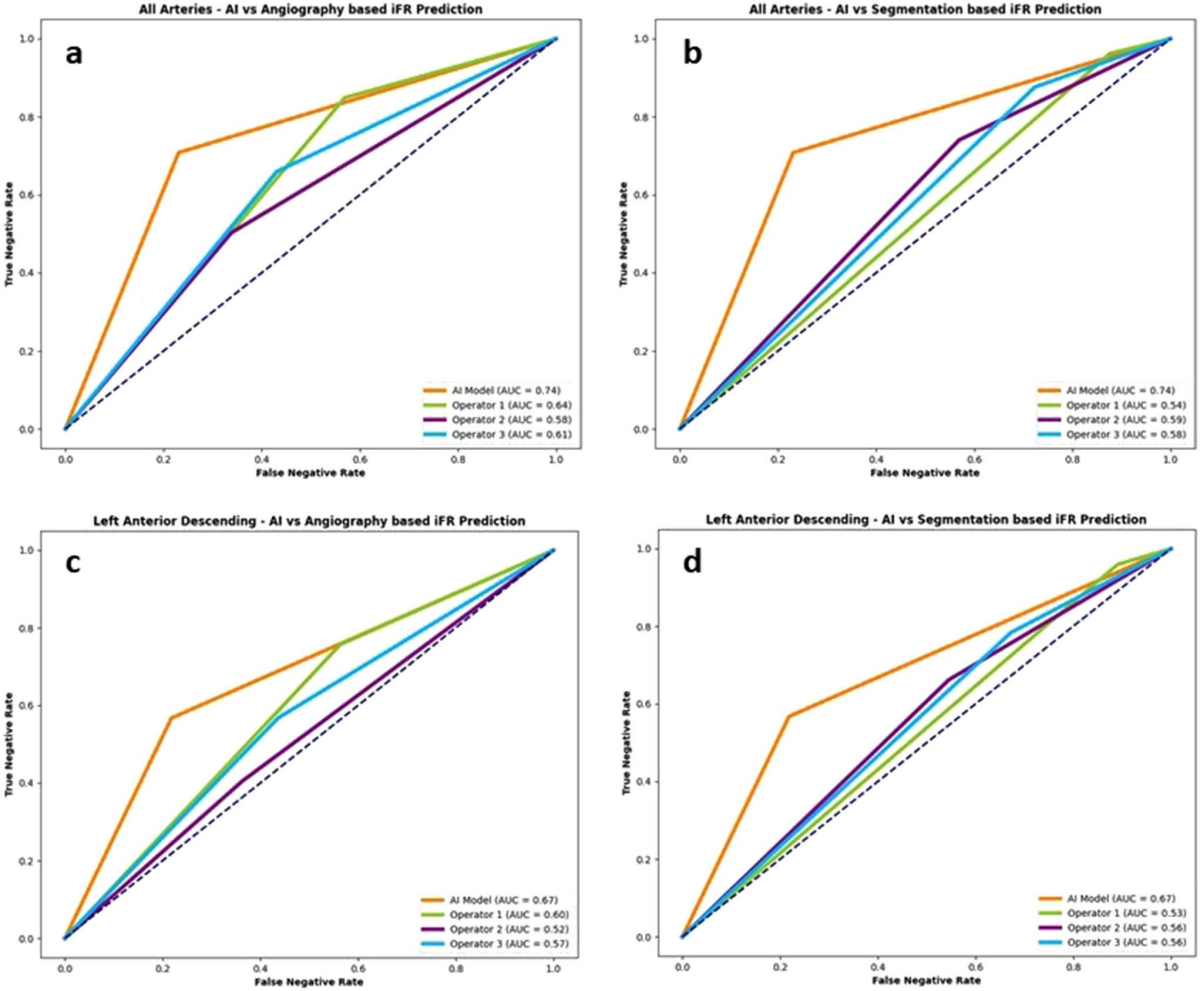
ROC curve analyzes for Artificial Intelligence (AI) and operators performance for all arteries and the left anterior descending artery (LAD). **(a)** AI and operators performance based on raw angiographic images for all arteries (AI AUC = 0.74; operators AUC = 0.58–0.64); **(b)** AI and operators performance based on segmented angiographic images for all arteries (AI AUC = 0.74; operators AUC = 0.54–0.59); **(c)** AI and operators performance based on raw angioraphic images for LAD (AI AUC = 0.57; operators AUC = 0.52–0.60); **(d)** AI and operators performance based on segmented angiographic images for LAD (AI AUC = 0.67; operators AUC = 0.55–0.56)

The comparison of IFR classification of lesions as measured *versus* as classified per each operator in raw angiography images is presented in Table 3. Regarding all the arteries analysis, the AI model and the operators accuracy, PPV, NPV, sensitivity and specificity are presented in Fig. 2. The difference between measured iFR classification and that predicted by interventional cardiologists was statistically significant for the three operators (*p* < 0.0001, *p* = 0.0012 and *p* = 0.02). The AUC in the modified ROC curve analysis (Fig. 3a) was superior for the AI measurements (AUC = 0.74), when compared to that of interventional cardiologists (AUC = 0.58–0.64).

**Table 3:**
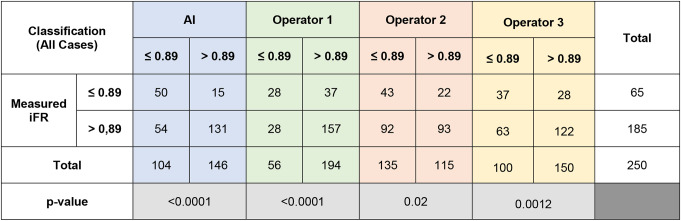
iFR classification of lesions as measured vs. as per each operator prediction for all cases

The comparison of IFR classification of lesions as measured versus as classified per each operator in segmented CAG analysis is presented in Supplementary data - Table 1 and accuracy, PPV, NPV, sensitivity and specificity are detailed in Fig. 2. Regarding segmented CAG, the difference between measured iFR classification and that predicted by interventional cardiologists was statistically significant for all the operators (*p* = 0.01, *p* = 0.004 and *p* = 0.0098). When comparing IFR classification based on CAG images versus segmented images, there was also a significant difference for the three operators (*p* < 0.0001). The AUC was once again inferior for operators as compared to AI (AUC = 0.54–0.59) - Fig. 3b.

### Left anterior descending artery (LAD)

**Fig. 4:**
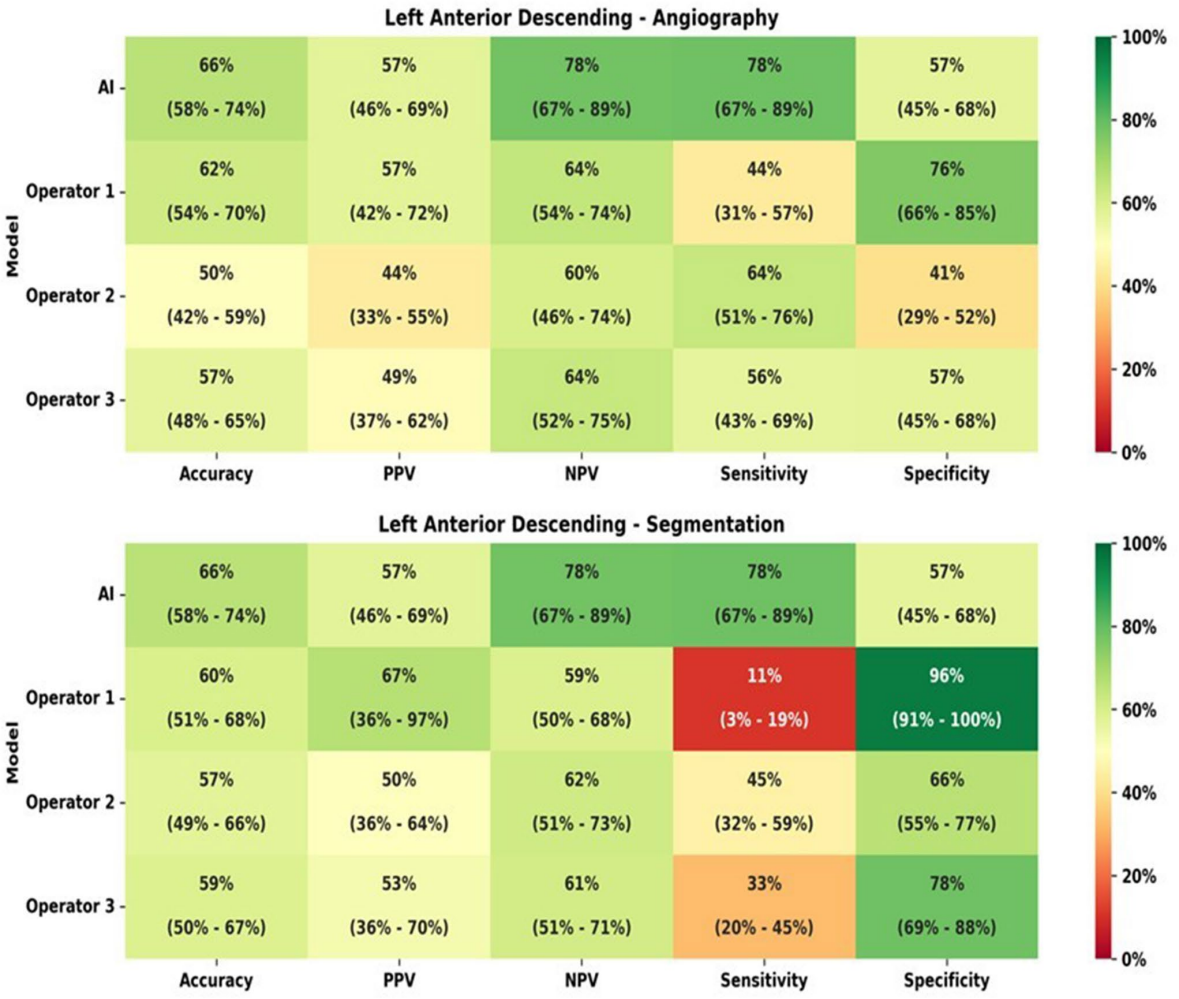
Heatmap analyzes for left anterior descending artery (LAD), regarding both angiography images and segmentation

The comparison of IFR classification of lesions as measured *versus* as classified per each operator with raw angiogram images is presented in Table 4 and the AI and the operators accuracy, PPV, NPV, sensitivity and specificity are detailed in Fig. 4.

**Table 4:**
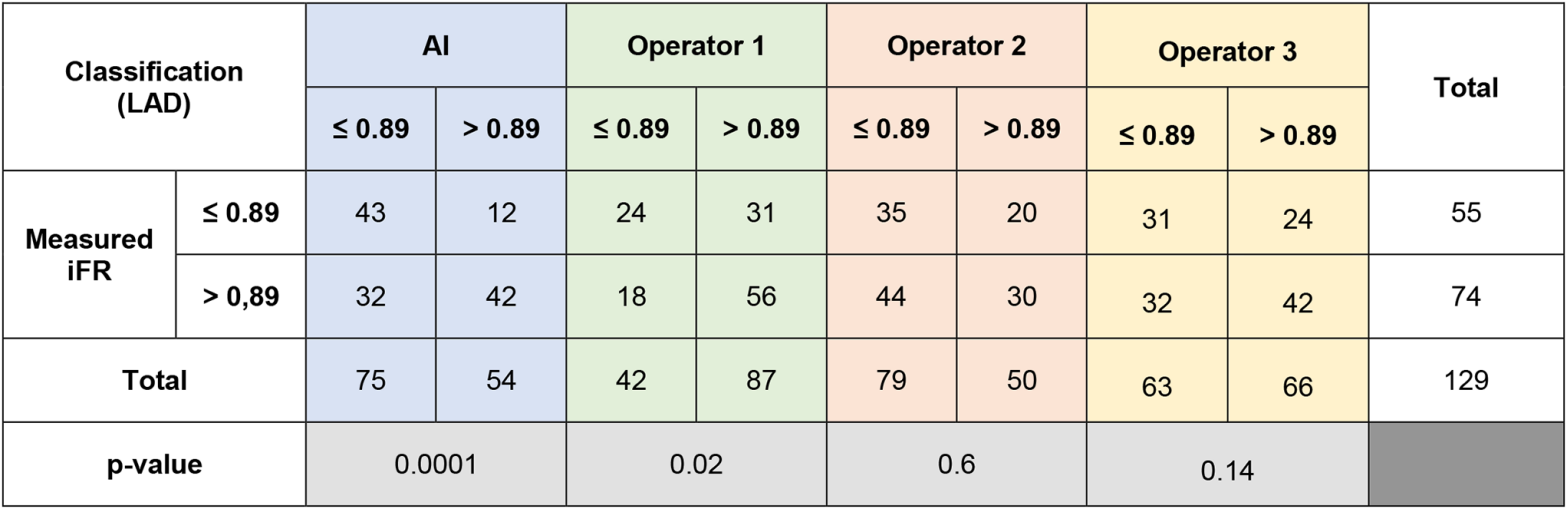
iFR classification of lesions as measured vs. as per each operator prediction for left anterior descending (LAD) cases

The difference between measured iFR classification and that predicted by interventional cardiologists was statistically significant for only one operator (*p* = 0.02, *p* = 0.14 and *p* = 0.6). The AUC was 0.67 for AI and ranged from 0.52 to 0.60 for the Cardiologists (Fig. 3c).

**Fig. 5:**
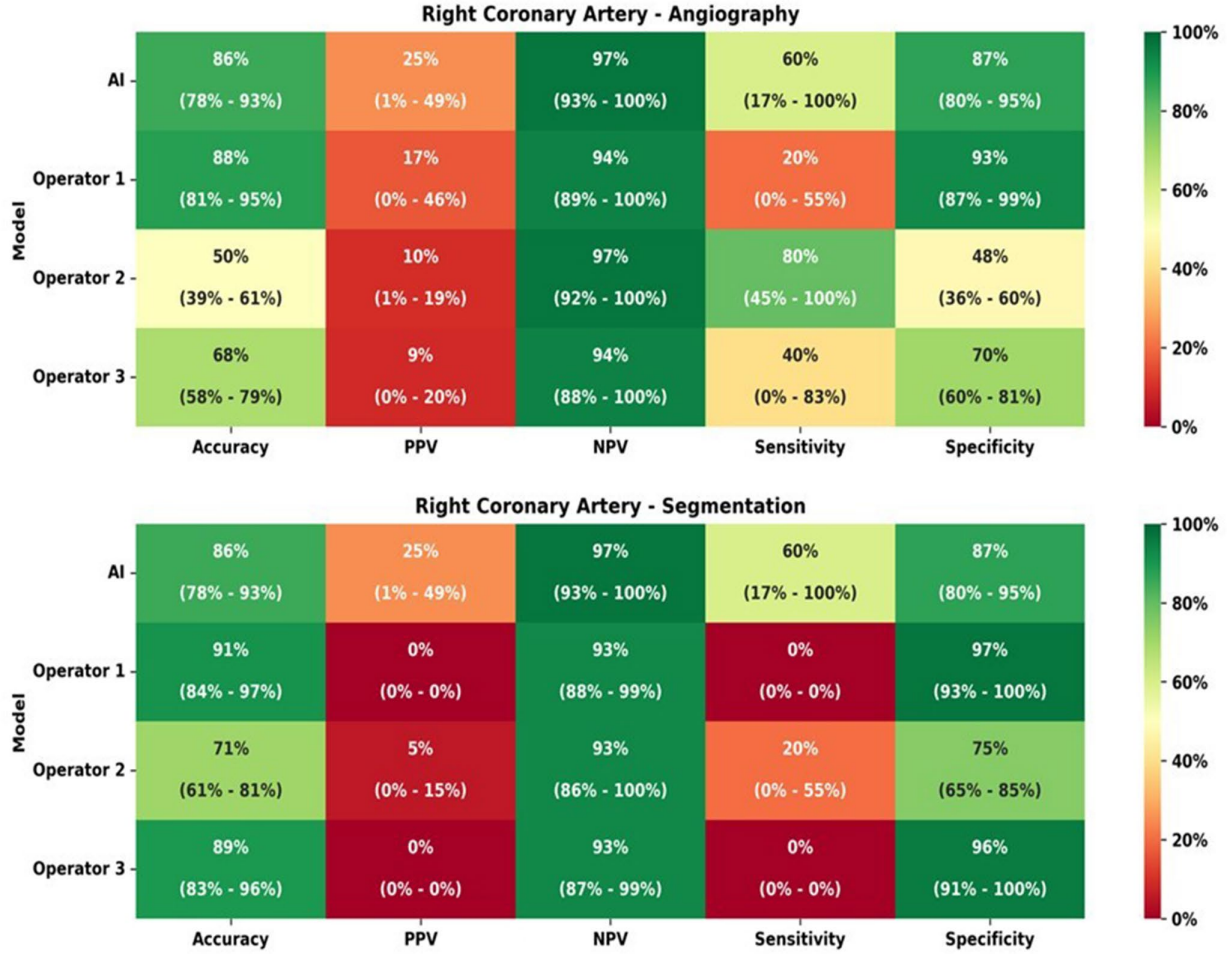
Heatmap analyzes for right coronary artery (RCA), regarding both angiography images and segmentation

In segmented CAG analysis, the comparison of IFR classification of lesions as measured *versus* as classified per each operator is presented in Supplementary data– Table 2 and accuracy, PPV, NPV, sensitivity and specificity are detailed in Fig. 5, as well as AUC in Fig. 3d.

Regarding segmented CAG, the difference between measured iFR classification and that predicted by interventional cardiologists was not statistically significant (*p* = 0.13, *p* = 0.16 and *p* = 0.18). However, when comparing IFR classification based on CAG images versus segmented images, there was a significant difference for the three operators (*p* = 0.0027, *p* < 0.0001 and *p* < 0.001).

### Right coronary artery (RCA)

The comparison of IFR classification of lesions as measured *versus* as classified per each operator with raw angiographic images is presented in Table 5 and accuracy, PPV, NPV, sensitivity and specificity are detailed in Fig. 5.

**Table 5:**
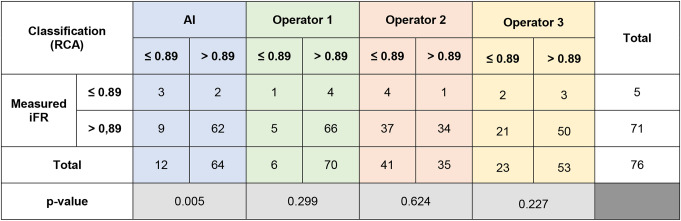
iFR classification of lesions as measured vs. as per each operator prediction for right coronary artery (RCA) cases

**Fig. 6:**
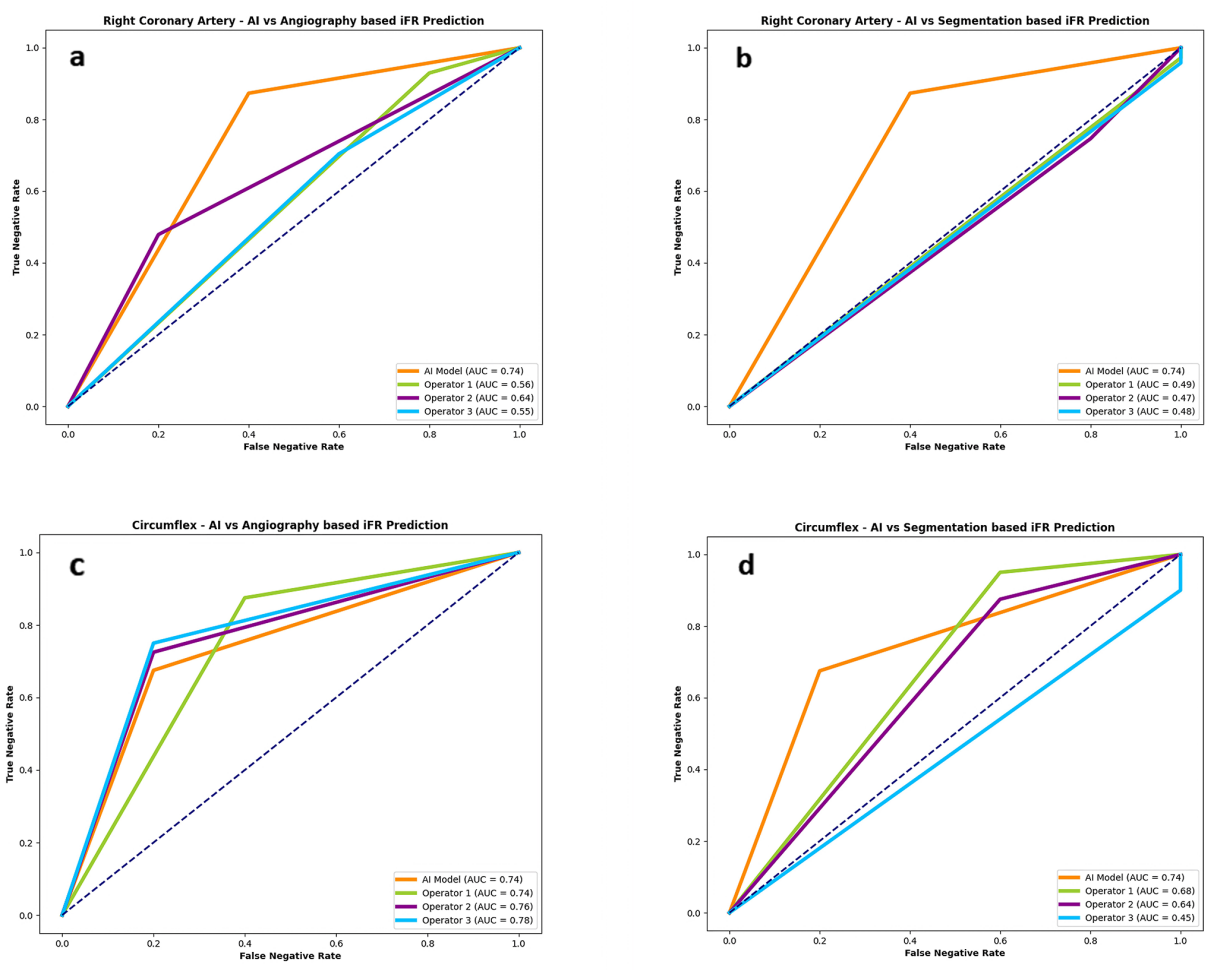
ROC curve analyzes for Artificial Intelligence (AI) and operators performance for the right coronary artery (RCA) and the Circumflex (Cx). **(a)** AI and operators performance based on raw angiographic images for the RCA (AI AUC = 0.74; operators AUC = 0.56–0.64); **(b)** AI and operators performance based on segmented angiographic images for the RCA (AI AUC = 0.74; operators AUC = 0.47–0.49); **(c)** AI and operators performance based on raw angioraphic images for the Cx artery (AI AUC = 0.74; operators AUC = 0.74–0.78); **(d)** AI and operators performance based on segmented angiographic images for the Cx artery (AI AUC = 0.74; operators AUC = 0.45–0.68)

The difference between measured iFR classification and that predicted by interventional cardiologists was not statistically significant (*p* = 0.299, *p* = 0.624 and *p* = 0.227). The AUC in the modified ROC curve analysis (Fig. 6a) was superior in AI measurements (AUC = 0.74), when compared to interventional cardiologists (AUC = 0.55–0.64).

The comparison of IFR classification of lesions as measured versus as classified per each operator in segmented CAG analysis is presented in Supplementary data– Table 3 and accuracy, PPV, NPV, sensitivity and specificity are detailed in Fig. 5. The operators’ AUC (Fig. 6b) was quite lower (0.47–0.49) than that of AI (AUC 0.74). Regarding segmented CAG, the difference between measured iFR classification and that predicted by interventional cardiologists was not statistically significant (*p* = 0.704, *p* = 0.639 and *p* = 0.789). However, when comparing IFR classification based on CAG images versus segmented images, there was a significant difference for the three operators (*p* < 0.0001, *p* = 0.0073 and *p* = 0.0463.

### Circumflex artery (Cx)

**Fig. 7:**
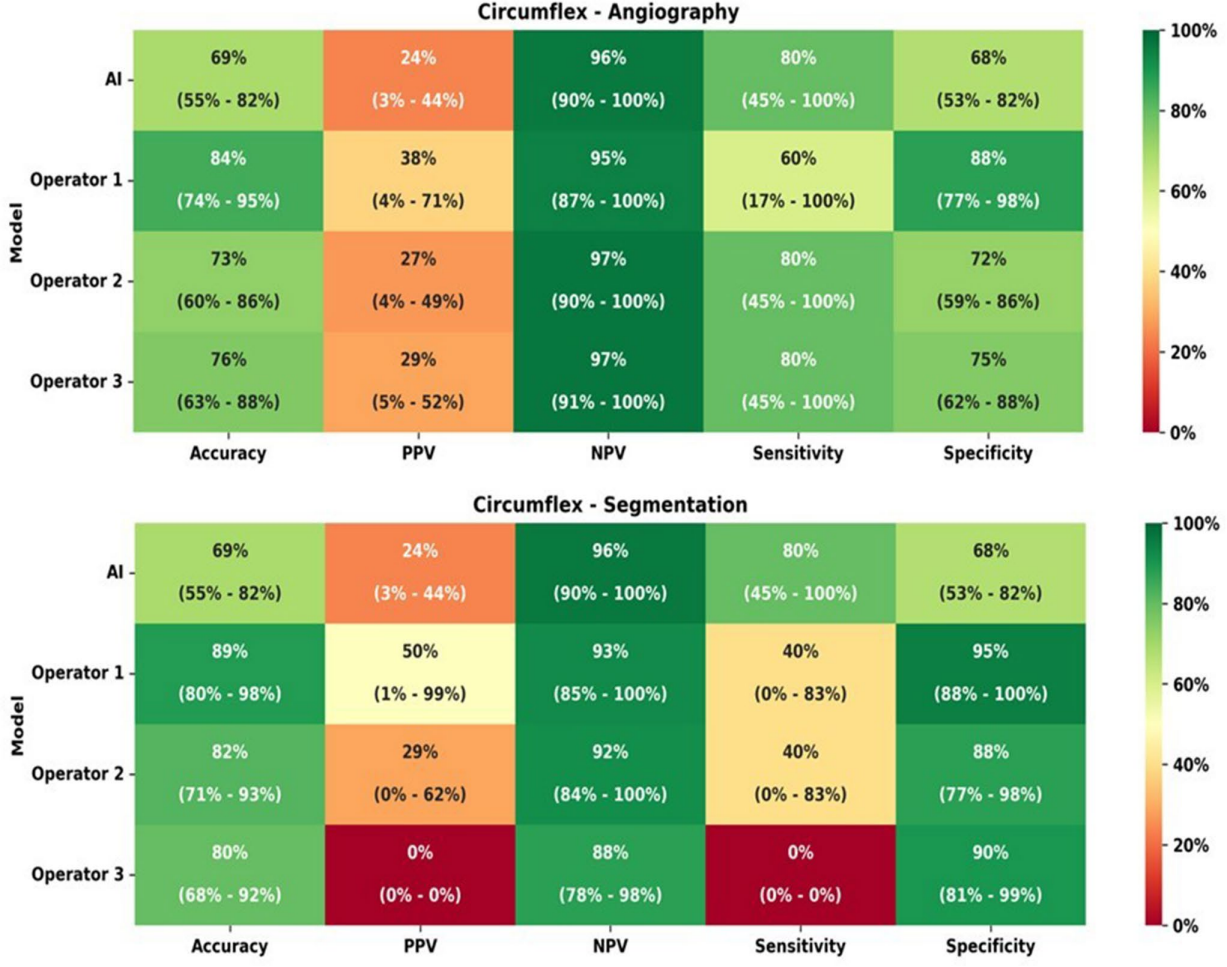
Heatmap analyzes for Circumflex artery (Cx), regarding both angiography images and segmentation

The comparison of IFR classification of lesions as measured *versus* as classified per each operator with raw angiographic images is presented in Table 6. The AI and the operators’ accuracy, PPV, NPV, sensitivity and specificity are detailed in Fig. 7.

**Table 6:**
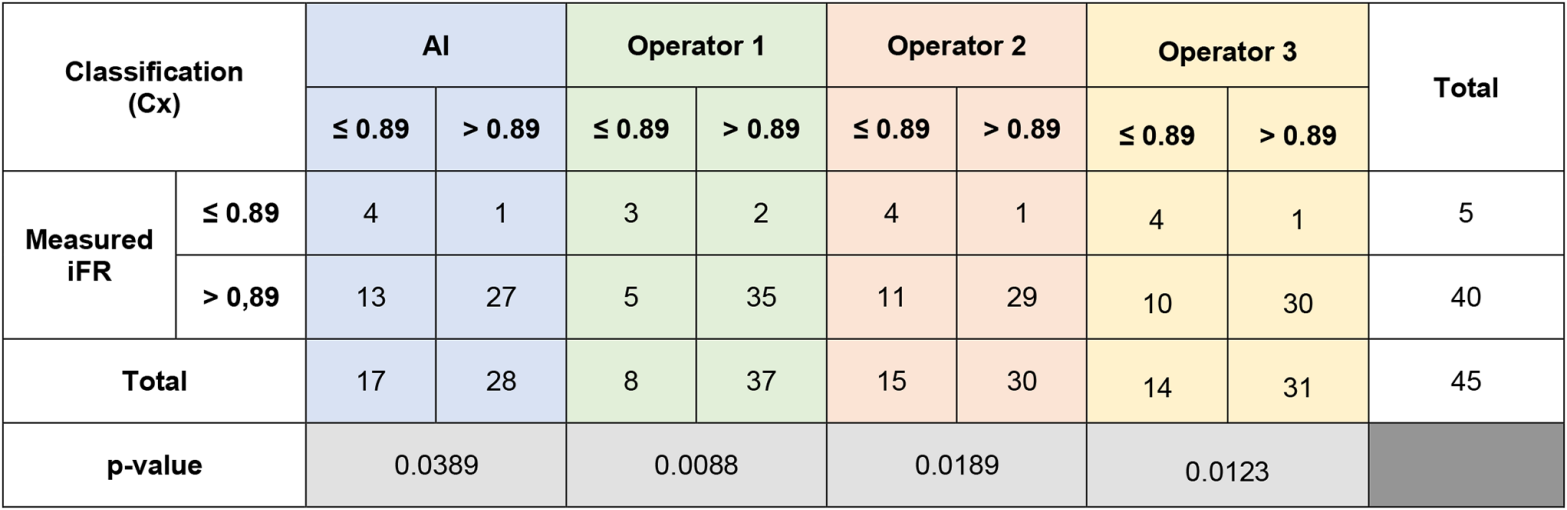
iFR classification of lesions as measured vs. as per each operator prediction for circumflex artery (Cx) cases

The difference between measured iFR classification and that predicted by interventional cardiologists was statistically significant for the three operators (*p* = 0.0088, *p* = 0.0123 and *p* = 0.0189). The AUC of AI was slightly inferior (0.74) to that of the operators (0.74 to 0.78) (Fig. 6c).

In segmented CAG analysis, the comparison of IFR classification of lesions as measured *versus* as classified per each operator is presented in data– Table 4 and and accuracy, PPV, NPV, sensitivity and specificity are detailed in Fig. 7. Regarding segmented CAG, the difference between measured iFR classification and that predicted by interventional cardiologists was statistically significant only for one operator (*p* = 0.0095, *p* = 0.46 and *p* = 0.11). When comparing IFR classification based on CAG images versus segmented images, there was a significant difference for two operators (*p* = 0.6922, *p* = 0.0470 and *p* = 0.0014).

The operators’ AUC (Fig. 6d) was once again inferior (0.45–0.68) comparing AI (0.45–0.68), albeit to a lesser degree than in the RCA.

## Discussion

We developed a fully automatic AI model capable of providing IFR binary classification derived from CAG images alone [37]. Despite the modest accuracy, the high negative predictive value (NPV of 90% in all arteries analysis) is clinically meaningful, as it offers the potential of terminating a diagnostic procedure without engaging in actual physiology measurements. This was especially relevant for the Cx and the RCA, given the NPV of 96% and 97%, respectively. In this paper, we aimed to find whether our AI method was superior, inferior or comparable to human performance, under similar conditions.

When comparing AI with the interventional cardiologists performance, there was considerable inter-operator and inter-artery heterogeneity, thereby significantly limiting very clear-cut general conclusions. However, the AI’s performance was generally mildly superior to that of operators, with mostly superior values of AUC and accuracy.

Interestingly, the AI model largely mimicked the operators’ strengths and weaknesses, in the sense that both had modest accuracy and AUC, high NPV, low PPV and low to modest sensitivity and specificity. This may partly be the result of a lesion dataset comprised mostly of lesions with an iFR > 0.89, as is typical of such populations [37]. This significantly limits both human and AI capability of identifying and correctly classifying positive lesions. It may also reflect intrinsic limitations of classifying iFR from CAG single frames alone. Notwithstanding, it is worth noting that AI had superior results regarding both PPV and sensitivity in almost all cases, thereby suggesting superior performance when dealing with positive (i.e. iFR < 0.89) lesions. This is of note, considering the limited amount of iFR < 0.89 cases available for training, as well as the much larger amount of exposure human operators have had as operators.

There were considerable differences when individual vessels were considered.

For the LAD, the general considerations above broadly apply, although attenuated: the negative predictive value was not as high, accuracy and AUC were lower, but both PPV and sensitivity were not as low. Generally, AI slightly outperformed operators.

For the RCA and Cx, both AI and operators were able to deal well with iFR negative (> 0.89) lesions, which were correctly classified very frequently. AI was more frequently correct when classifying a lesion as negative (i.e., higher NPV). Human operators were better capable of identifying negative lesions (i.e. higher specificity), especially in the circumflex artery. In both arteries, both AI and human operators incorrectly classified lesions as positive very frequently, but AI was more sensitive to positive lesions than humans, once more suggesting that it may have greater potential in dealing with such lesions, despite the limited number of cases available for training.

Segmentation had a mixed effect on operator classification of lesions. In all arteries, particularly the RCA and Cx, it led to a much greater degree of negative classifications and a lower tendency to classify lesions as positive. As a result, specificity was increased at the expense of sensitivity. And while the PPV was slightly improved in the LAD (where the majority of positive cases were concentrated), the opposite ensued for the remaining arteries, where PPV was very low, sometimes reaching 0. Lastly, NPV was slightly reduced by segmentation as well. The AUC plots clearly reflect this trade-off, as the increased capacity for identifying negatives is substantially affected by the tendency to overclassify lesions as negative, resulting in lower AUCs and thus a lower discriminatory ability to truly detect negative lesions. These findings are likely the result of the effect segmentation has in reducing the operators’ lesion severity estimation, as we have previously demonstrated [37]. While that may be of use in an all-comers population (as in the one we have previously published) [37], significantly reducing the tendency for classifying non-severe lesions as severe, in a selected population of intermediate lesions, it seems segmentation somewhat overcorrects this tendency and is largely not advantageous.

Multiple randomized trials have proven the benefit of physiology-guided PCI, significantly reducing death, myocardial infarction and repeat revascularization [4, 5, 7]. More recently, a randomized trial comparing FFR versus angiography-guided strategy in acute myocardial infarction with multivessel disease [38], showed that a FFR-guided decision making was superior to a strategy of angiography only-guided PCI for treatment of non-infarct-related artery lesions, when considering a composite of time to death, MI or repeat revascularization.

Despite the established evidence, coronary physiology remains largely underused [17, 18]. Some challenges associated with the technicalities of the procedure and the risk of procedure complications due to guiding-catheters and guide-wires use may explain why [17, 18]. Coronary angiography digitally-derived index (either FFR or iFR) could bypass this limitations and has been explored in recent years, with commercial softwares made recently available. Most studies were based on FFR, using a ≤ 0.80 threshold. The FAST-FFR [21] was a multicenter international study of 301 patients with a predominant LAD lesion (54.2%). When comparing the measured and the estimated FFR (FFRangio), the accuracy was 92.2%, the NPV 94.8%, the PPV 89%, the sensitivity 93.5% and the specificity 91.2% [21]. The correlation between the measured and the FFRangio was *r* = 0.80 (*p* < 0.001) [21]. Witberg et al. performed a pooled analysis of 5 cohort studies, with similar results [39]. More studies have shown encouraging results of FFRangio [20, 40] and the iFR from CAG has also been explored on the REVEAL iFR trial [41], with published results expected soon. These studies used mostly non-AI methods to derive from CAG, using a combination of three dimensional image reconstruction and computational fluid dynamics. Although they demonstrated that physiology can successfully be derived from CAG images alone, they are not without caveats: significant manual input is required, more than one projection may be required and inter-operator heterogeneity has been reported, with inferior performance to that of a core-lab [42].

Although AI have shown great potential in Cardiovascular Medicine, it’s use in coronary physiology is still under development. Three studies of FFR estimation from CAG using primarily AI methods were published [33, 43, 44]. Roguin et al. conducted a pilot study using a novel automated artificial intelligence angiography-based FFR software, capable of conducting a binary analysis of FFR ≤ 0.80 [33]. In a single center population consisting of 31 patients with predominantly LAD lesions (80%), they reported an accuracy of 90%, a NPV of 87%, a PPV of 94%, sensitivity of 88% and specificity of 93% when conducting a binary analysis of FFR ≤ 0.80 [33]. Their single model is able to derive an estimated FFR value, with an area under the curve of 0,91 and an r correlation coefficient of 0,71 (*p* < 0.001) [33].

Cho et al. [43] used a very large sample of 1501 lesions from a single center (predominantly LAD– 67%) and plotted the target vessel diameters together with clinical characteristics (age, sex, body surface area, and target segment) to binarily classify FFR measurements with a ≤ 0.80 threshold. An overall accuracy of 82%, a NPV of 84%, a PPV of 81%, sensitivity of 84% and specificity of 80% were reported in the test set, similarly to the external validation dataset of 79 patients [43]. However, their methodology is semi-automatic, since it requires significant manual annotations.

*Arefinia et al.* [44] designed a deep learning model for estimating the value of FFR using angiographic images to classify LAD stenosis between 50 and 70%. 3625 images were extracted from 41 patients’ angiographic films and FFR was also classified as negative (FFR > 0.80) and positive (≤ 0.80). An AUC, accuracy, sensitivity, specificity and positive predictive value of 0.81, 0.81, 0.86, 0.75 and 0.82, respectively, were described. Despite their impressive metrics, the very small sample size significantly limits analysis, given that only a small pool of measurements is available for testing. and the fact that they omitted the potential influence of factors such age and gender in FFR estimation. External validation is also needed.

### Limitations

Given the heavy reliance on large volumes of data for AI training, the size of our dataset emerged as an important limitation. This was particularly significant for cases involving the Cx and RCA where iFR was ≤ 0.89. Nonetheless, our distribution of target vessels and positive/negative cases aligns with previously reported data [11, 15, 20, 21, 33, 40]. Consequently, a much larger dataset for effective training is required, securing a dataset with a sufficient number of iFR-positive cases, particularly for the RCA and Cx.

Although a 10-fold cross-validation approach is a standard practice in machine learning, it’s use can be considered a limitation. An 80/20% train/test split would have led to a limited testing set, hindering our ability to accurately assess model performance, especially in analyzing performance across different target vessels due to the dataset’s inherent imbalance. To overcome this limitation, we used a 10-fold cross-validation, as described.

FFR has been directly compared to angiography in clinical outcomes studies [4–6], while iFR has only been compared directly to FFR [11, 12, 15]. Extrapolating iFR instead of FFR can be pointed as another potential limitation. However, iFR has consistently proven to be non-inferior to FFR and has become the preferred tool for assessing epicardial physiology in many laboratories due to its simplicity. As a result, the number of iFR measurements in our lab significantly surpasses those of FFR, offering a more extensive foundation for future training, refinement, and validation.

Another limitation lies in the model’s current ability for binary classification. During initial testing, it became evident that estimating the precise iFR value would demand a substantially larger dataset, which exceeded the scope of this initial study.

The use of a single frame for model training, rather than a 3D reconstruction based on multiple 2D projections, was also a limitation, considering the inherently 3D nature of coronary arteries and the superior performance of non-AI approaches utilizing 3D methods. The interventional cardiologists were exposed to a single “raw” fluoroscopic frame, thereby mimicking what the AI models actually “see”. However, this approach is considerable different from the daily clinical practice, which may have compromised their performance. Furthermore, because operators have access to a full cine loop, it’s possible that human performance would be different had we provided them that. However, the comparison would then be unbalanced, given that we would effectively be giving humans more complete data than to AI models, which may significantly limit the interpretation of results.

Another limitation relies on the fact that this was a single-center retrospective dataset and future external validation will be necessary. However, given that physiology results have been shown to be quite reproducible, the impact of this particular limitation is likely to be minimal.

The fact that unsuccessful segmentation was used as a selection criteria may limit generalizability due to selection bias. However, we have previously shown that our segmentation AI model is accurate and capable, and in this study failed segmentation was a result of either poor image quality or artifacts (such as pacemaker leads, surgery stiches, etc.). We therefore do not believe this will be a significant limitation going forward.

Therefore, this study is exploratory, serving as a proof of concept. We intend to significantly expand the training dataset through multi-institutional collaboration, which will be crucial for improving performance and enabling external validation. We also plan to enhance our models by incorporating multiple projections and 3D reconstruction, which could potentially boost performance. Ultimately, our goal is to deploy this for clinical use, as either new software or an enhancement of current non-AI methods.

## Conclusion

We developed an AI model capable of binary iFR classification of lesions. Despite modest overall performance, AI mildly outperformed experienced interventional cardiologists, despite a much smaller exposure to CAG images than that of humans. When dealing with negative lesions, both AI and humans performed well. AI was superior in correctly classifying negative lesions (i.e. higher NPV), but human operators proved more capable of detecting such lesions (i.e. higher specificity). Positive lesions were very often missed (i.e. low sensitivity) or incorrectly classified (i.e. low PPV), although less so with AI.

Despite not yet mature enough for clinical deployment, our latest data builds on previously published studies, highlighting the potential of AI in improving and accelerating the performance of coronary lesions assessment, hopefully contributing to the future implementation of AI as a diagnostic aid in cath labs, as well as increasing the adoption of physiology (by its virtualization). Larger datasets and future validation studies are necessary for clinical deployment. But even if only a consistently very high negative predictive value is achieved at an earlier development stage, one could arguably integrate it into existing workflows by ending the procedure straight away, rather than engaging if invasive measurements.

## Electronic supplementary material

Below is the link to the electronic supplementary material.

Supplementary Material 1

## Data Availability

No datasets were generated or analysed during the current study.

## Abbreviations

ACS: Acute Coronary Syndrome
AI: Artificial Intelligence
AUC: Area Under the Curve
CABG: Coronary Artery Bypass Surgery
CAD: Coronary Artery Disease
CAG: Coronary Angiography
Cx: Circumflex Artery
FFR: Fractional Flow Reserve
FN: False Negative (FN)
FP: False Positive (FP)
iFR: instantaneous Free-Wave Ratio
LAD: Left Anterior Descending Artery
NPV: Negative Predictive Value
PCI: Percutaneous Coronary Intervention
PPV: Positive Predictive Value
RCA: Right Coronary Artery
STEMI: ST Segment Elevation Myocardial Infarction
TP: True Positive (TP)
TN: True Negative (TN)
