# Supplementary material for: Non-invasive derivation of instantaneous free-wave ratio from invasive coronary angiography using a new deep learning artificial intelligence model and comparison with human operators’ performance
Source: Int J Cardiovasc Imaging. 2025 Mar 10;41(4):755–71. doi: 10.1007/s10554-025-03369-y (PMC11982120; doi:10.1007/s10554-025-03369-y)
Supplement: Supplementary file 1 — Supplementary Material 1 [file 10554_2025_3369_MOESM1_ESM.docx]

**Supplementary data**

| **Classification**  **(All Cases)** | | **AI** | | **Operator 1:**  **segmentation** | | **Operator 2:**  **segmentation** | | **Operator 3:**  **segmentation** | | **Total** |
| --- | --- | --- | --- | --- | --- | --- | --- | --- | --- | --- |
|  |  | **≤ 0.89** | **> 0.89** | **≤ 0.89** | **> 0.89** | **≤ 0.89** | **> 0.89** | **≤ 0.89** | **> 0.89** |  |
| **Measured iFR** | **≤ 0.89** | 50 | 15 | 8 | 57 | 28 | 37 | 18 | 47 | 65 |
|  | **> 0,89** | 54 | 131 | 7 | 178 | 48 | 137 | 23 | 162 | 185 |
| **Total** | | 104 | 146 | 15 | 235 | 76 | 174 | 41 | 209 | 250 |
| **p-value** | | <0.0001 | | 0.0128 | | 0.0043 | | 0.0098 | |  |

Table 1: iFR classification of lesions as measured vs as per each operator prediction for all cases in segmented CAG analysis.

| **Classification**  **(LAD)** | | **AI** | | **Operator 1:**  **segmentation** | | **Operator 2:**  **segmentation** | | **Operator 3:**  **segmentation** | | **Total** |
| --- | --- | --- | --- | --- | --- | --- | --- | --- | --- | --- |
|  |  | **≤ 0.89** | **> 0.89** | **≤ 0.89** | **> 0.89** | **≤ 0.89** | **> 0.89** | **≤ 0.89** | **> 0.89** |  |
| **Measured iFR** | **≤ 0.89** | 43 | 12 | 6 | 49 | 25 | 30 | 18 | 37 | 55 |
|  | **> 0,89** | 32 | 42 | 3 | 71 | 25 | 49 | 16 | 58 | 74 |
| **Total** | | 75 | 54 | 9 | 120 | 50 | 79 | 34 | 95 | 129 |
| **p-value** | | 0.0001 | | 0.1307 | | 0.1568 | | 0.1785 | |  |

Table 2: iFR classification of lesions as measured vs as per each operator prediction for left anterior descending (LAD) cases in segmented CAG analysis.

| **Classification**  **(RCA)** | | **AI** | | **Operator 1:**  **segmentation** | | **Operator 2:**  **segmentation** | | **Operator 3:**  **segmentation** | | **Total** |
| --- | --- | --- | --- | --- | --- | --- | --- | --- | --- | --- |
|  |  | **≤ 0.89** | **> 0.89** | **≤ 0.89** | **> 0.89** | **≤ 0.89** | **> 0.89** | **≤ 0.89** | **> 0.89** |  |
| **Measured iFR** | **≤ 0.89** | 3 | 2 | 0 | 5 | 1 | 4 | 0 | 5 | 5 |
|  | **> 0,89** | 9 | 62 | 2 | 69 | 18 | 53 | 3 | 68 | 71 |
| **Total** | | 12 | 64 | 2 | 74 | 19 | 57 | 3 | 73 | 76 |
| **p-value** | | 0.0050 | | 0.7037 | | 0.6391 | | 0.7894 | |  |

Table 3: iFR classification of lesions as measured vs as per each operator prediction for right coronary artery (RCA) cases in segmented CAG analysis.

| **Classification**  **(Cx)** | | **AI** | | **Operator 1:**  **segmentation** | | **Operator 2:**  **segmentation** | | **Operator 3:**  **segmentation** | | **Total** |
| --- | --- | --- | --- | --- | --- | --- | --- | --- | --- | --- |
|  |  | **≤ 0.89** | **> 0.89** | **≤ 0.89** | **> 0.89** | **≤ 0.89** | **> 0.89** | **≤ 0.89** | **> 0.89** |  |
| **Measured iFR** | **≤ 0.89** | 4 | 1 | 2 | 3 | 2 | 3 | 0 | 5 | 5 |
|  | **> 0,89** | 13 | 27 | 2 | 38 | 5 | 35 | 4 | 36 | 40 |
| **Total** | | 17 | 28 | 4 | 41 | 7 | 38 | 4 | 41 | 45 |
| **p-value** | | 0.0389 | | 0.0095 | | 0.4588 | | 0.1097 | |  |

Table 4: iFR classification of lesions as measured vs as per each operator prediction for circumflex artery (Cx) cases in segmented CAG analysis.

**Supplementary data legends**

Table 1: iFR classification of lesions as measured vs as per each operator prediction for all cases in segmented CAG analysis.

Table 2: iFR classification of lesions as measured vs as per each operator prediction for left anterior descending (LAD) cases in segmented CAG analysis.

Table 3: iFR classification of lesions as measured vs as per each operator prediction for right coronary artery (RCA) cases in segmented CAG analysis.

Table 4: iFR classification of lesions as measured vs as per each operator prediction for circumflex artery (Cx) cases in segmented CAG analysis.
